# Receptor of Advanced Glycation End Products Deficiency Attenuates Cisplatin-Induced Acute Nephrotoxicity by Inhibiting Apoptosis, Inflammation and Restoring Fatty Acid Oxidation

**DOI:** 10.3389/fphar.2022.907133

**Published:** 2022-05-30

**Authors:** Qiang Wang, Yuemei Xi, Binyang Chen, Hairong Zhao, Wei Yu, De Xie, Weidong Liu, Furong He, Chenxi Xu, Jidong Cheng

**Affiliations:** ^1^ Department of Internal Medicine, Xiang’an Hospital of Xiamen University, School of Medicine, Xiamen University, Xiamen, China; ^2^ Xiamen Key Laboratory of Translational Medicine for Nucleic Acid Metabolism and Regulation, Xiamen, China

**Keywords:** rage, cisplatin-induced nephrotoxicity, apoptosis, inflammation, fatty acid oxidation

## Abstract

Cisplatin is a widely used and potent anti-neoplastic agent, but severe and inescapable side effects in multiple normal tissues and organs limit its application, especially nephrotoxicity. Molecular mechanisms of cisplatin nephrotoxicity involve mitochondrial damage, oxidative stress, endoplasmic reticulum stress, inflammation, apoptosis, necroptosis, etc. Receptor of advanced glycation end products (RAGE) is a multiligand pattern recognition receptor, engaged in inflammatory signaling and mitochondrial homeostasis. Whether inhibition of RAGE alleviates cisplatin-induced nephropathy has not been investigated. Here, we revealed that RAGE deficiency attenuates cisplatin-induced acute nephrotoxicity, as evidenced by reduced apoptosis, inflammation, lipid accumulation, restored mitochondrial homeostasis and fatty acid oxidation in renal tubular epithelial cells (TECs). *In vitro* studies showed that, the RAGE-specific inhibitor FPS-ZM1 attenuated the cisplatin-induced decrease of cell viability and fatty acid oxidation in the normal rat renal TEC line NRK-52E cells. Taken together, RAGE knockout mitigated cisplatin-induced acute nephrotoxicity by inhibiting apoptosis, inflammation, and restoring fatty acid oxidation in TECs, suggesting that RAGE inhibition could be a therapeutic option for cisplatin-induced acute nephrotoxicity.

## Introduction

Acute kidney injury (AKI) is a prevalent complication in hospitalized patients with an incidence of 10–15% (Ronco et al., 2019). Cisplatin is a frequently utilized and effective chemotherapeutic agent in clinical settings, which renders it a common cause of AKI. The exploration of the molecular mechanisms of cisplatin nephrotoxicity has never halted; however, the mechanisms have yet been fully defined, so therapies that can abolish cisplatin-induced AKI always remain lacking (McSweeney et al., 2021). Studies have demonstrated that cisplatin nephrotoxicity links to mitochondrial damage, oxidative stress, endoplasmic reticulum stress, inflammation, apoptosis, necroptosis (Xu et al., 2015; Zahedi et al., 2017; Lu et al., 2020; Mapuskar et al., 2021), etc. According to recent reports, impaired fatty acid oxidation (FAO) plays a key role in the process of cisplatin nephrotoxicity (Chiba et al., 2019; Jang et al., 2020). What’s more, compromised FAO in renal TECs is considered an essential pathogenesis of renal interstitial fibrosis (Kang et al., 2015).

RAGE is a multiligand pattern recognition receptor implicated in inflammatory signaling (Coughlan et al., 2009; Adeshara et al., 2018). Meanwhile, RAGE modulates glucose and lipid metabolism, e.g., senescence-induced RAGE promotes hepatic steatosis by suppressing FAO (Song et al., 2014; Wan et al., 2020). It was found that RAGE blockade could relieve tubular and glomerular damage resulting from diabetes as well as glomerulosclerosis induced by adriamycin (Guo et al., 2008; Matsui et al., 2017; Sanajou et al., 2019). However, whether RAGE inhibition can alleviate cisplatin-induced AKI, mainly characterized by TEC injury, and its underlying mechanisms remain unexplored. Thus, we used RAGE global knockout (RAGE-/-) mice to pursue the role of RAGE in cisplatin-induced AKI *in vivo*; moreover, we revealed the role of RAGE-specific inhibitor FPS-ZM1 in cisplatin-induced NRK-52E cellular insult *in vitro* and aimed to further dissect the potential molecular mechanisms.

## Materials and Methods

### Reagents

Cisplatin (#T1564) is from Topsicence (United States); FPS-ZM1 (#HY-19370) is from MedChemExpress (United States); rabbit anti-RAGE (#ab3611) is from Abcam (United Kingdom); rabbit anti-Bax (#CPA1092), and Bcl-2 (#CPA1095) antibodies are from Cohesion Bioscience (United Kingdom); rabbit anti-Phospho-NF-κB p65 (Ser536) (#3033) and NF-κB p65 (#3034) antibodies are from Cell Signaling Technology (United States); rat anti-F4/80 antibody (#14-4801-82) is from Invitrogen United States; mouse anti-GAPDH (#AC033), rabbit anti-β-actin (#AC026), Cpt1a (#A5307) and PGC-1α (#A12348) and horseradish peroxidase (HRP)-conjugated goat anti-rabbit IgG (#AS014) antibodies are from ABclonal (China). One Step Terminal transferase dUTP nick-end labelling (TUNEL) Apoptosis Assay Kit (#C1090) is from Beyotime (China). SuperKine™ West Femto Maximum Sensitivity Substrate (#BMU102-CN) is from Abbkine (China).

### Cell Culture and Treatment

Normal rat renal TEC line NRK-52E is from Center for Excellence in Molecular and Cellular Sciences, Chinese Academy of Sciences (China), routinely cultured with DMEM (#PM150210, Procell, China) containing 5% fetal bovine serum in incubator of 37°C, 5% carbon dioxide. To evaluate the role of FPS-ZM1 in cisplatin-induced cellular insult, NRK-52E cells were pretreated with FPS-ZM1 alone for 6 h, followed by co-treatment with cisplatin for 24 h.

### Mice

RAGE-/- mice of C57BL/6J background were kindly shared by Prof. Ben Lu (Central South University, China) (Deng et al., 2018), and the genotypes of the mice was confirmed with real-time PCR and western blot (Supplementary Figure S1). All mice were kept at Xiamen University Laboratory Animal Center (XMULAC) under a 12-h light/dark cycle, provided with standard chow diet and water *ad libitum*.

Mice were randomized into four groups: 1) wild type (WT) mice receiving vehicle (0.5% sodium carboxymethylcellulose), 2) WT mice receiving cisplatin (dissolved in 0.5% sodium carboxymethylcellulose to form a homogeneous suspension, 20 mg/kg body weight, single intraperitoneal injection), 3) RAGE-/- mice receiving vehicle, and 4) RAGE-/- mice receiving cisplatin. Mice were sacrificed 72 h following cisplatin administration. All mice for experiments were male, SPF grade, 6–8 weeks weighting 18∼22 g. All procedures were approved by the Animal Care and Use Committee of Xiamen University.

### Renal Function Assessment

Renal function is indicated by serum creatinine (CREA) and blood urea nitrogen (BUN), both of which are measured by fully automated biochemistry analyzer (#BS-240VET, Mindray, China).

### Oil Red O Staining

Frozen sections of renal tissue were maintained in Oil Red O working solution at room temperature for 1 h, washed 3 times with double distilled water, and re-stained with hematoxylin. After being rinsed with tap water, the sections were mounted with glycerol gelatin, observed and photographed under a light microscope (#DM2700 P, Leica, Germany).

### Cell Counting Kit-8 Assay

Cells were seeded in 96-well plates (8,000 cells, 100 μl medium per well). After allowed to adhere overnight, cells were pretreated with FPS-ZM1 for 6 h followed by co-treatment with cisplatin for 24 h. Finally, 10 μl CCK-8 solution was added to each well, and the absorbance at 450 nm was detected after 2-h incubation.

### Quantitative Real-Time PCR

Total RNA was extracted and purified using RNA extraction kit with mini spin columns (#AG21022, Accurate Biology, China), 2 mg of which was then subject to reverse transcription with Evo M-MLV Mix Kit (#AG11728, Accurate Biology, China). Relative quantification of mRNA was performed on a Real-Time PCR Detection System (#CFX96, Bio-Rad, United States) using SYBR Green Mix (#AH0104-B, SparkJade Biotechnology, China), and then calculated by 2−ΔΔCT method. Primer sequences are listed in Supplementary Table S1.

### Western Blot

Western blot assay was performed as previously described (Wang et al., 2021). Briefly, protein lysates from cells or mouse renal cortex were harvested with radioimmunoprecipitation assay buffer (#EA0002, SparkJade Biotechnology, China). Aliquots were electrophoretically separated by 10∼13% SDS-PAGE and transferred to PVDF membranes (#BSP0161, PALL, United States), which were incubated at 4°C overnight with proper primary antibodies followed by HRP-labeled secondary antibody at room temperature for 1 h. Finally, images were captured by Azure C280 system (United States) and analyzed with ImageJ software (National Institutes of Health, United States).

### Transmission Electron Microscopy

Briefly, 1 mm^3^ upper pole renal cortex was sequentially harvested and immobilized in 2.5% neutral glutaraldehyde fixative and 1% osmium acid at 4°C. Tissues were dehydrated in gradient acetone and then embedded, followed by cut into 50 nm ultrathin sections using ultramicrotome. Finally, sections were double stained with uranyl acetate and lead nitrate. Transmission electron microscope (#HT-7800, Hitachi, Japan) was used for mitochondrial observation and image acquisition.

### Assessment of Tubular Damage

Fresh murine kidneys were paraffin-embedded after immobilization in 4% paraformaldehyde, and then cut into 5-μm-thick sections for subsequent hematoxylin-eosin (HE) staining and Periodic Acid Schiff (PAS) staining. Tubular injury in PAS-stained sections was rated *as per* the proportion of damage area: 0 = normal, 1 = 1∼25, 2 = 26∼50, 3 = 51∼75, and 4 = 76∼100%. Finally, TUNEL fluorescence staining was carried out for renal tubular apoptotic cell evaluation according to the manufacturer’s instructions.

### Immunohistochemical Staining of F4/80

Immunohistochemistry was performed as previously described (Zou et al., 2016). Briefly, fresh kidney tissues were embedded in paraffin and sectioned into 5 μm following immobilization in paraformaldehyde solution. Then, the sections were deparaffinized, hydrated, antigen-repaired, and endogenous peroxidase activity was abrogated by 3% hydrogen peroxide. Rat anti-F4/80 antibody (1:200) and goat HRP-conjugated anti-rat secondary antibody (1:200) were used in sequence for section incubation. Finally, antigen of F4/80 was localized by chromogenic substrate of DAB working solution.

### Statistical Analysis

Date are expressed as mean ± standard error of the mean (SEM) and calculated with One-way or Two-way analysis of variance (ANOVA) when comparing means between groups in the GraphPad Prism software (version 9.0.0, United States). *p* < 0.05 was considered statistically significant.

## Results

### RAGE Products Knockout Attenuated Cisplatin-Induced Nephropathy

**FIGURE 1:**
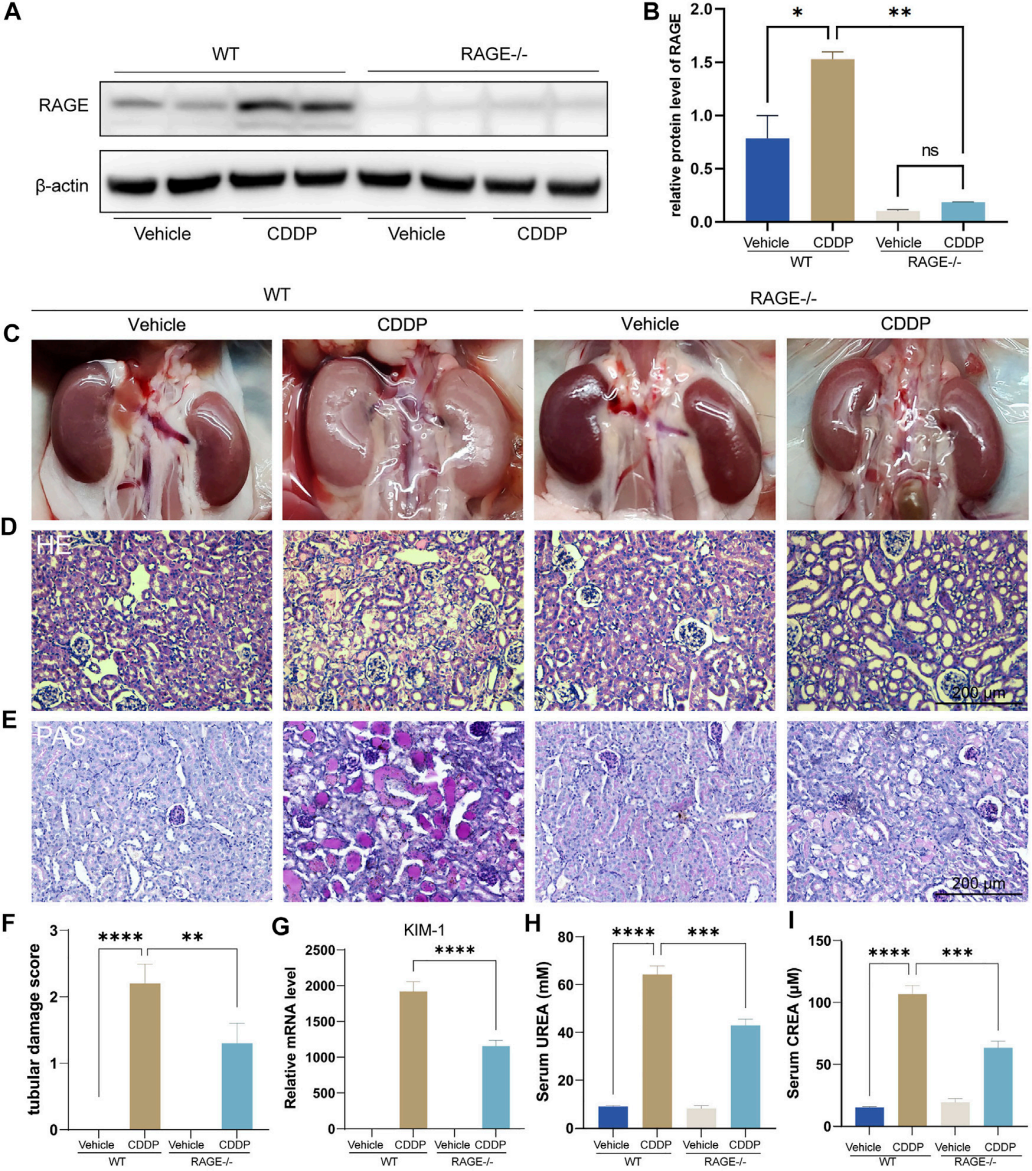
RAGE knockout attenuated cisplatin-induced nephropathy. The protein level of RAGE was detected by western blot **(A)** and semi-quantified in a histogram **(B)**. **(C)** Representative macroscopic murine kidneys. **(D)** HE staining of paraffin sections of mouse kidneys. **(E)** PAS staining and **(F)** corresponding tubular injury scores of paraffin sections of mouse kidney. **(G)** Detection of mRNA level of KIM-1, a marker of kidney injury by qPCR. **(H,I)** Determination of UREA and CREA levels in mice. One-way ANOVA was used to compare means between groups. Data are expressed as mean ± SEM. Scare bar: 200 μm. **p < 0.01, ***p < 001, ****p < 0.0001. WT, wild type; CDDP, cisplatin.

We first assessed if RAGE blockade could attenuate cisplatin-induced kidney injury using RAGE knockout mice. Mice were subject to intraperitoneal injection of a single dose of cisplatin (20 mg/kg). After 72 h, blood and kidneys were harvested for subsequent examinations. Western blot showed that cisplatin significantly upregulated the protein level of renal RAGE, which was eliminated by RAGE knockout (Figures 1A,B). The macroscopic kidneys of WT mice receiving cisplatin appeared grayish white, indicating the notable nephrotoxicity of cisplatin. The macroscopic kidneys of RAGE-/- mice receiving cisplatin showed significant attenuation of renal graying, suggesting that RAGE knockout may ameliorate the cisplatin-induced nephrotoxicity (Figure 1C). Consistently, HE and PAS staining showed dramatic tubular necrosis triggered by cisplatin, whereas deletion of RAGE significantly diminished the lesion (Figures 1D,E), which was semi-quantified in Figure 1F. Meanwhile, we detected canonical markers of renal injury and discovered that cisplatin significantly upregulated the mRNA level of KIM-1 in kidney, serum concentrations of creatinine (CREA) and blood urea nitrogen (BUN), while RAGE knockout partially reversed these markers (Figures 1G–I). In summary, RAGE knockout attenuated cisplatin-induced renal injury in mice.

### RAGE Products Knockout Reduced Cisplatin-Induced Apoptosis of Nephrocytes

**FIGURE 2:**
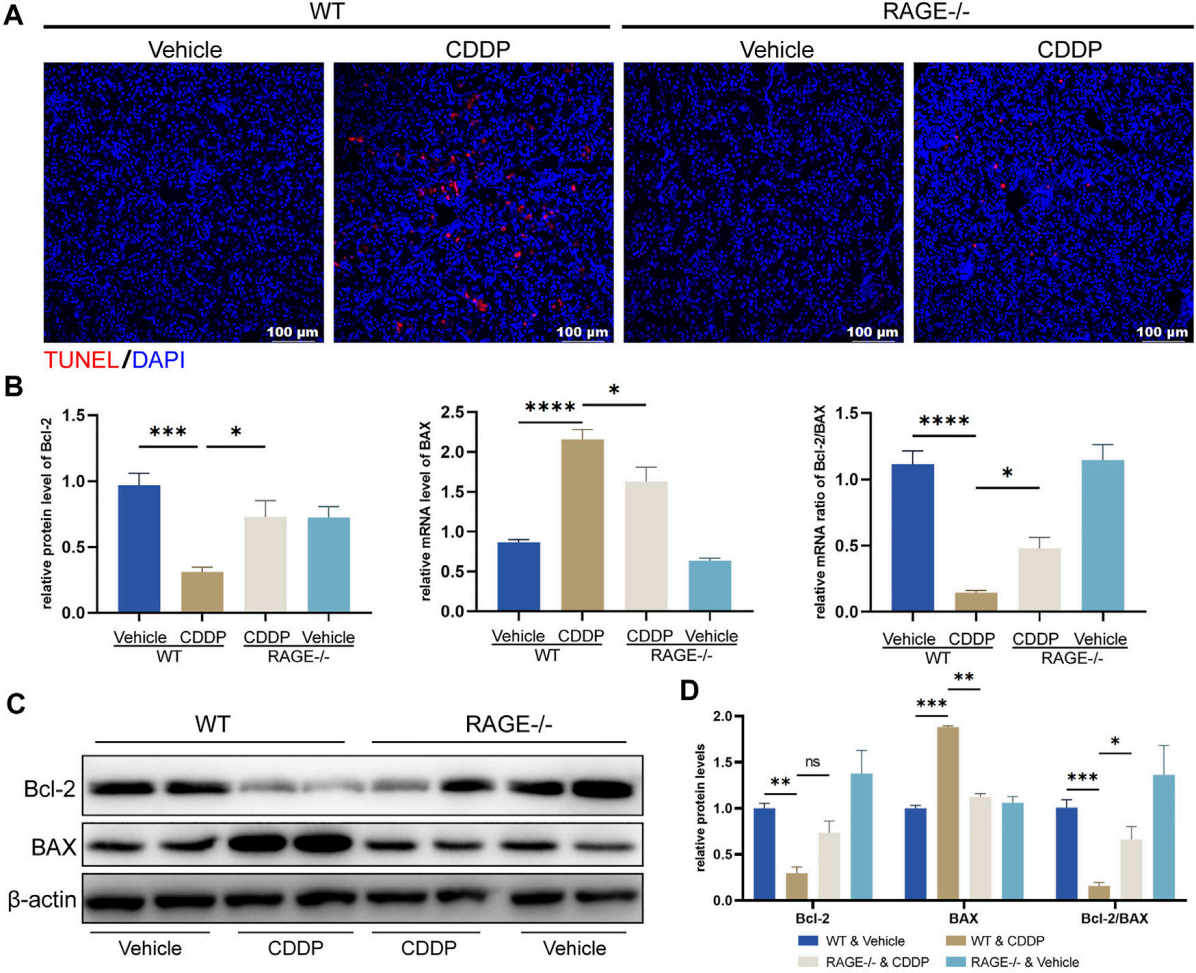
RAGE knockout reduced cisplatin-induced apoptosis of nephrocytes. **(A)** TUNEL staining of paraffin sections of mouse kidneys. **(B)** The mRNA levels of Bcl-2 and BAX were detected by qPCR, and the ratio of Bcl-2/BAX was calculated. One-way ANOVA was used to compare means between groups. **(C)** The protein levels of Bcl-2 and BAX were detected by western blot and then semi-quantified in a histogram with Two-way ANOVA **(D)**. Data are expressed as mean ± SEM. Scale bar: 100 μm. *p < 0.05, **p < 0.01, ***p < 001, ****p < 0.0001. ns, not significant.

Apoptosis of proximal renal tubules is an essential mechanism of cisplatin-induced nephrotoxicity (Kaushal et al., 2008; Ozkok and Edelstein, 2014; Zhang et al., 2017). To assess if RAGE knockout could exert an effect on cisplatin-induced tubular apoptosis, we performed TUNEL staining and examined mRNA and protein levels of apoptosis-related genes. The number of TUNEL-positive cells (red fluorescence) was significantly increased in WT mice subjected to cisplatin, which was attenuated by RAGE knockout (Figure 2A). Cisplatin decreased the mRNA level of anti-apoptotic gene Bcl-2, increased the mRNA level of pro-apoptotic gene BAX in WT mouse kidneys, and as a result, the Bcl-2/BAX ratio was downregulated, while RAGE knockdown reverted these modifications (Figure 2B). Consistently, RAGE knockout curtailed the overexpression of BAX protein triggered by cisplatin (Figure 2C). Although RAGE knockout failed to rescue the expression of downregulated anti-apoptotic protein Bcl-2, the decreased ratio of Bcl-2/BAX was still restored (Figure 2C). All these were semi-quantified in the Figure 2D. In conclusion, genetic blockade of RAGE reduced cisplatin-induced renal tubular apoptosis.

### RAGE Products Knockout Mitigated Cisplatin-Induced Renal Inflammation

**FIGURE 3:**
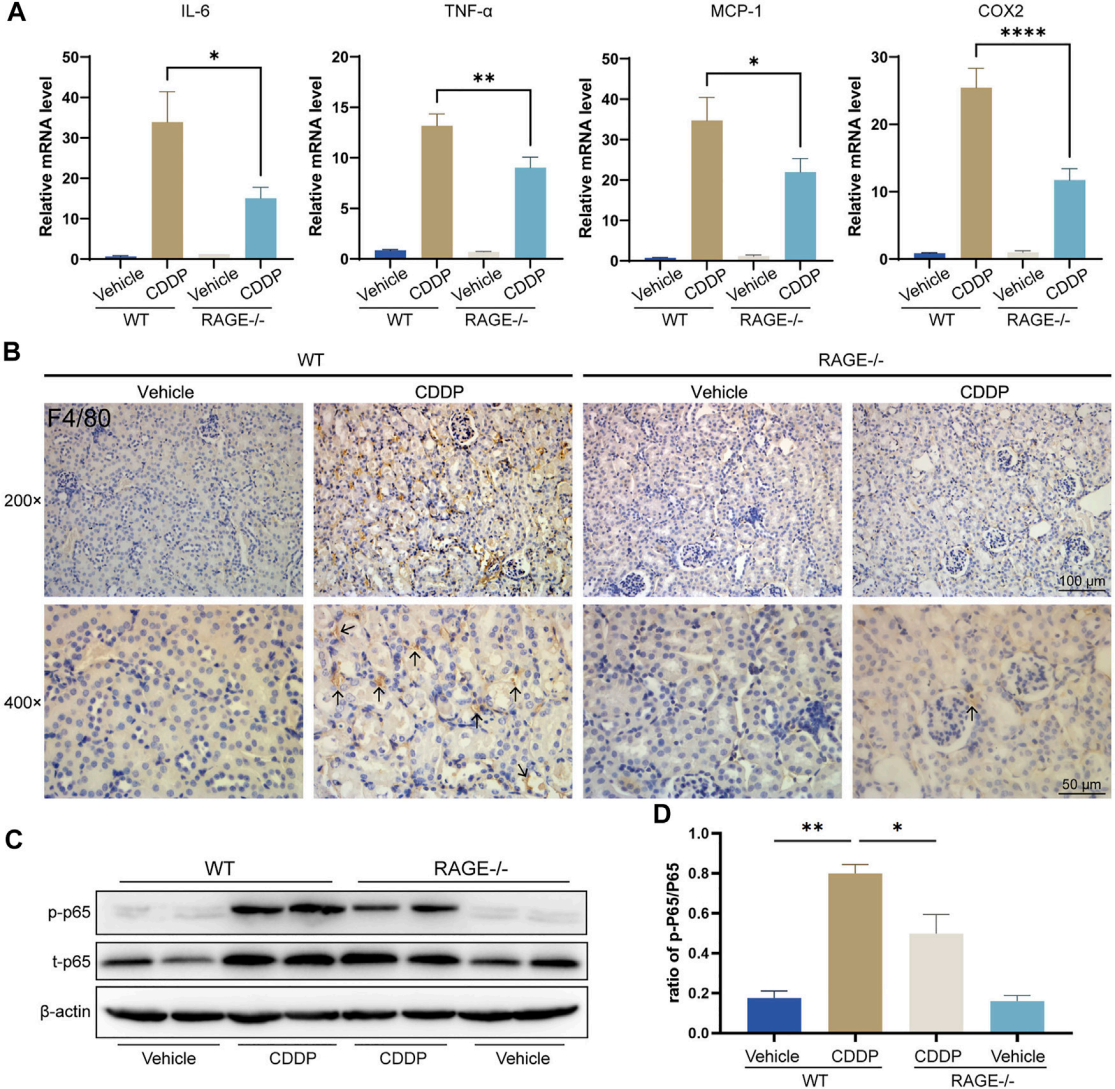
RAGE knockout mitigated cisplatin-induced renal inflammation. **(A)** qPCR detection of mRNA levels of inflammation-related indicators in mouse kidney, including pro-inflammatory factors IL-6 and TNF-α, chemokine MCP-1, and COX-2. **(B)** Immunohistochemical staining of mouse kidney paraffin sections. Brown marked by arrows means F4/80-positive, which indicates infiltration of macrophages. **(C)** The protein levels of phosphorylated p65 and total p65 were detected by western blot and then semi-quantified in a histogram **(D)**. One-way ANOVA was used to compare means between groups. Data are expressed as mean ± SEM. Scale bar: 100 or 50 μm as indicated in the figure. *p < 0.05, **p < 01, ****p < 0.0001. p, phosphorylated; t, total.

Renal inflammation response, especially immune cell infiltration, is a typical mechanism of cisplatin nephrotoxicity (Pabla and Dong, 2008; Manohar and Leung, 2018). To evaluate renal inflammation, we determined the relative transcript levels of pro-inflammatory factors and used immunohistochemistry to assess renal immune infiltration. The results showed that cisplatin markedly upregulated the transcript levels of tumor necrosis factor α (TNF-α), interleukin 6 (IL-6), monocyte chemoattractant protein-1 (MCP-1), and cyclooxygenase-2 (COX-2) in the renal cortex tissue and dramatically increased macrophage infiltration (F4/80-positive), while RAGE knockout significantly alleviated these alterations (Figures 3A,B). Given that NF-κB is a central element mediating cisplatin-induced renal inflammation (Ozkok et al., 2016; Yu et al., 2018), We further investigated the expression level of NF-κB p65, and the results showed that both p-p65 and total p65 were significantly upregulated in mice allocated cisplatin. However, overexpression of p-p65 was not fully due to the increase of total p65, because the ratio of p-p65 to total p65 still surged when cisplatin was administrated, which was partly but significantly quenched in RAGE knockout mice (Figures 3C,D). Taken together, RAGE suppression attenuated cisplatin-induced murine renal inflammation.

### RAGE Products Knockout Restored Cisplatin-Induced Mitochondrial Homeostasis

**FIGURE 4:**
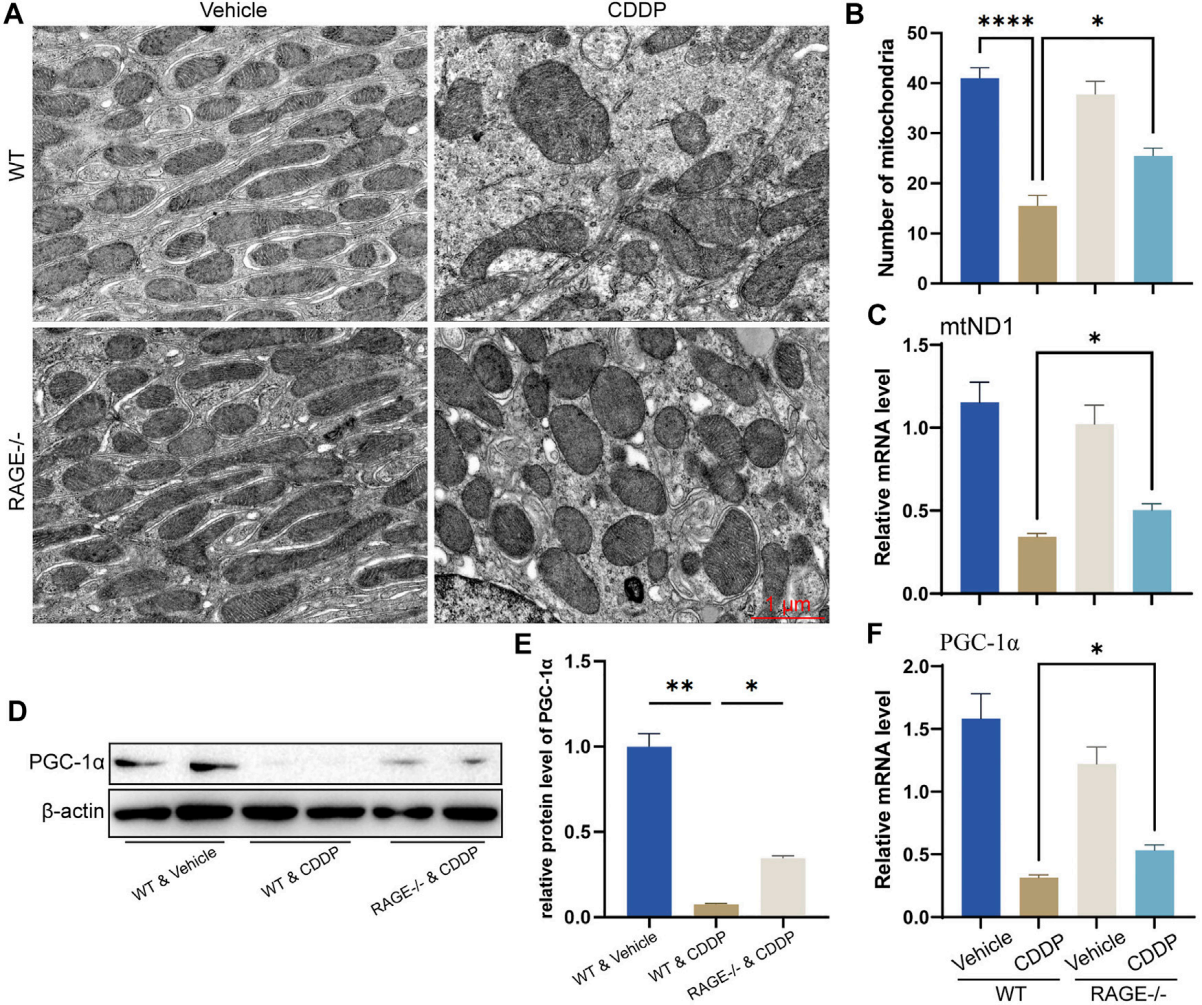
RAGE knockout restored cisplatin-induced mitochondrial homeostasis. **(A)** Mitochondrial morphology of mouse renal tubular epithelial cells was observed using transmission electron microscopy (TEM). **(B)** The number of mitochondria was counted according to TEM. **(C)** The mRNA level of mtND1, indicating the relative copy number of cellular mitochondria. **(D)** The protein level of PGC-1α was detected by western blot and then semi-quantified in a histogram **(E)**. **(F)** The mRNA level of PGC-1α determined by qPCR. One-way ANOVA was used to compare means between groups. Data are expressed as mean ± SEM. Scale bar: 1 μm. *p < 0.05, **p < 01, ****p < 0.0001.

Mitochondrial homeostasis is essential for the survival of renal TECs (Szeto, 2017; Wang et al., 2020). Cisplatin significantly disturbs the mitochondrial homeostasis of renal TECs, resulting in nephrotoxicity (Wang et al., 2018; Lu et al., 2020). To determine if RAGE deficiency restores mitochondrial homeostasis in the kidney of mice subject to cisplatin, we performed TEM. Mitochondrial morphology of renal TECs in RAGE-/- mice was comparable to that of WT mice. After cisplatin administration for 3 days, WT mice showed significant swelling and reduced number of mitochondria as well as disappearance of mitochondrial cristae (Figures 4A,B). Similar alterations were indicated by mitochondrial copy number according to the qPCR result (Figure 4C). Considering that PGC-1α is a central regulator of mitochondrial biosynthesis (Qian et al., 2019; Fontecha-Barriuso et al., 2020; Popov, 2020), we further examined its mRNA and protein levels and found that RAGE knockout rescued the cisplatin-induced PGC-1α suppression (Figures 4D–F). Collectively, RAGE knockout restored cisplatin-induced mitochondrial homeostasis.

### RAGE Products Knockout Diminished Cisplatin-Induced Renal Lipid Accumulation and FAO Impairment

**FIGURE 5:**
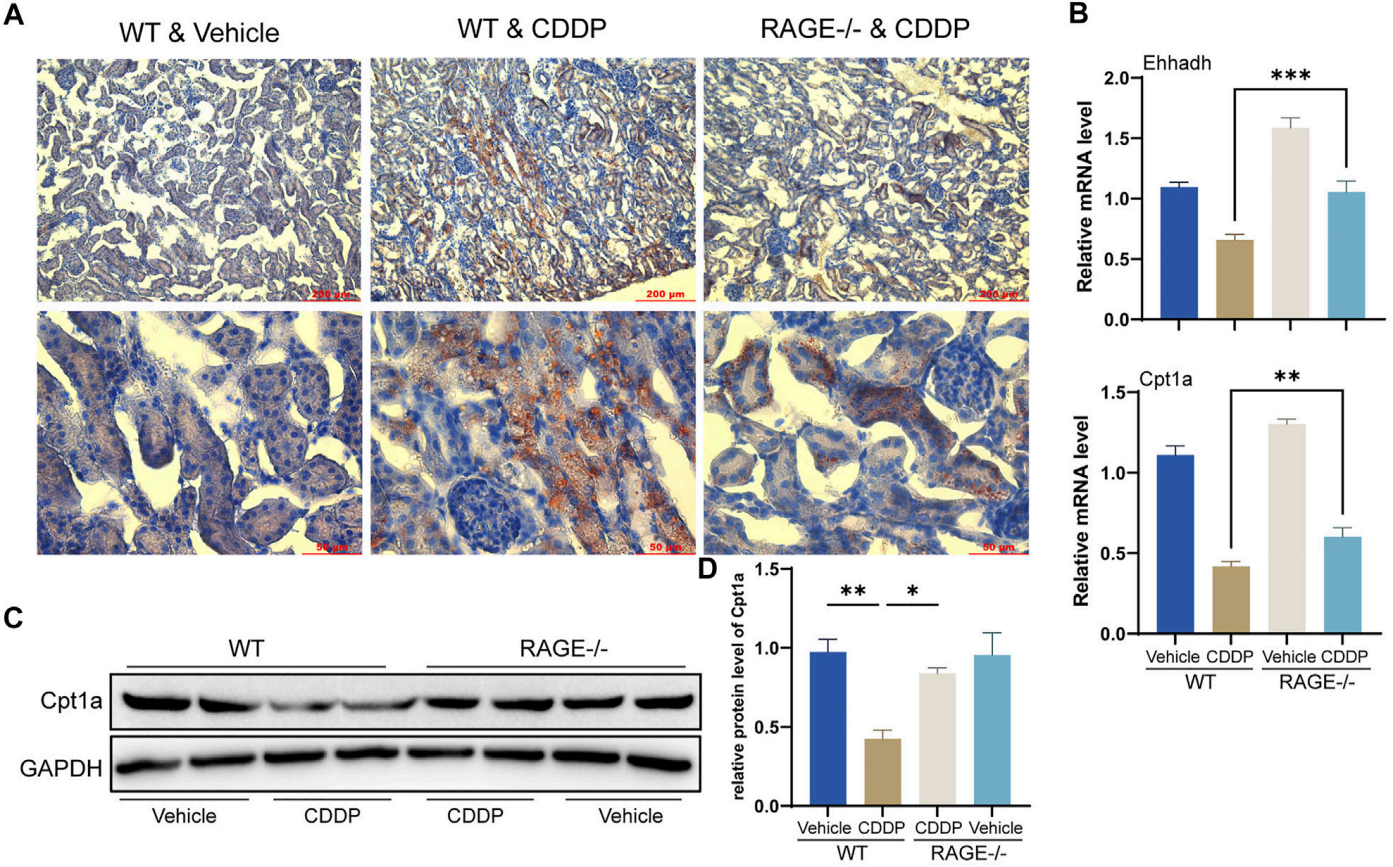
RAGE knockout diminished cisplatin-induced renal lipid accumulation and FAO impairment. **(A)** Oil Red O staining was performed to assess lipid deposition in the kidney tissues. **(B)** The histogram indicated mRNA levels of FAO-related genes Ehhadh and Cpt1a. **(C)** Cpt1a was selected for validation of protein expression with subsequent semi-quantification **(D)**. One-way ANOVA was used to compare means between groups. Data are expressed as mean ± SEM. Scale bar: 200 or 50 μm as indicated in the figure. *p < 0.05, **p < 0.01, ***p < 0.001.

FAO is the primary energy source for renal TECs (Kang et al., 2015). Cisplatin blocks this process, thereby preventing cellular access to energy and ultimately causing lipid accumulation and cell injury (Li et al., 2020). Oil Red O staining showed that TECs of WT mice receiving cisplatin exhibited significant lipid accumulation, while RAGE knockout significantly attenuated this alteration (Figure 5A). Further qPCR results revealed a significant reduction in transcription levels of FAO-related genes Ehhadh and Cpt1a in WT mice receiving cisplatin and a marked improvement in the RAGE-/- mice receiving cisplatin (Figure 5B). Cpt1a was taken for further validation at the protein level (Figures 5C,D). In summary, RAGE knockout diminished cisplatin-induced renal lipid accumulation and FAO impairment.

### The RAGE Products‐Specific Inhibitor FPS-ZM1 Restored Cisplatin-Suppressed NRK-52E Cell Viability

**FIGURE 6:**
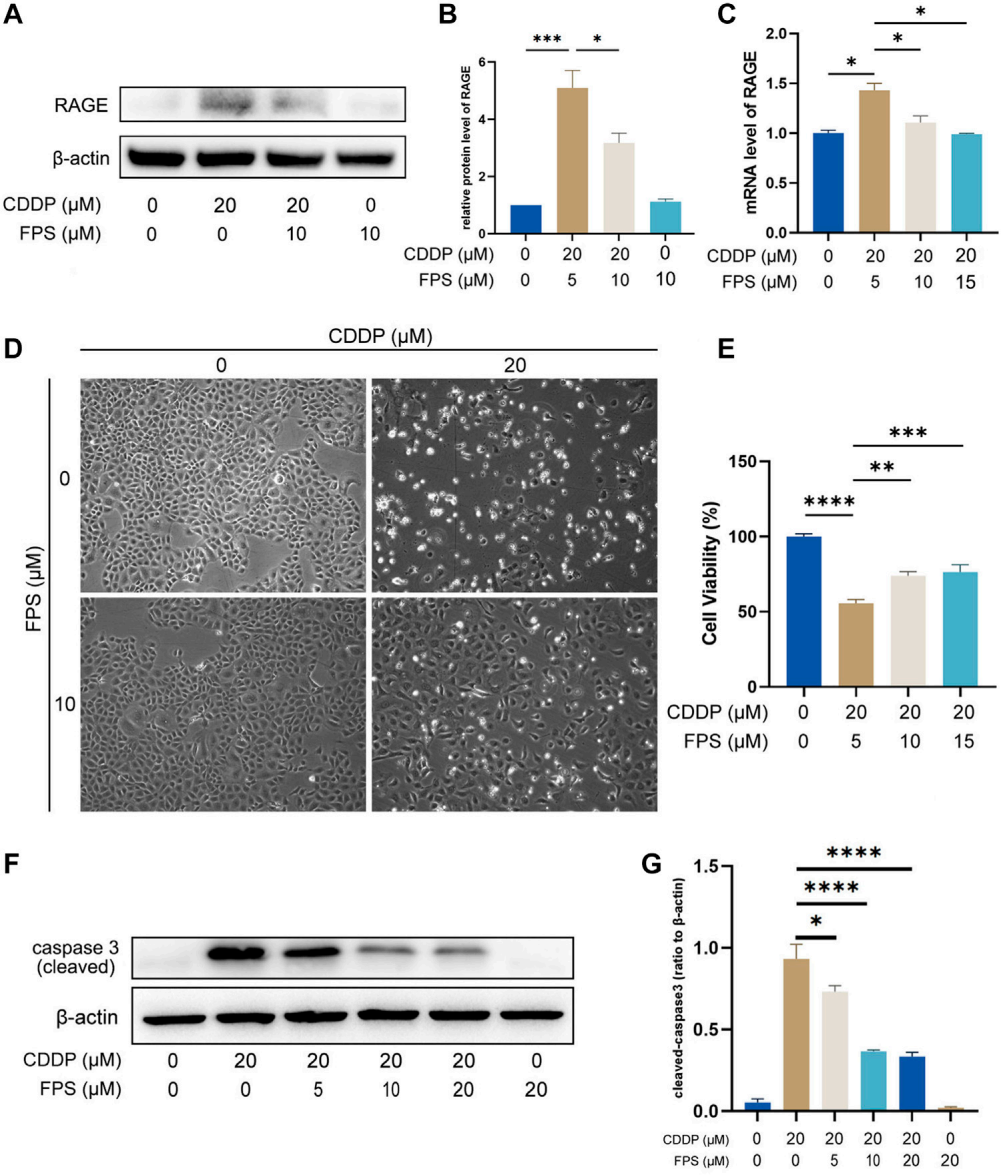
The RAGE-specific inhibitor FPS-ZM1 restored cisplatin-suppressed NRK-52E cell viability. The protein level of RAGE in NRK-52E cells was accessed by western blot **(A)** and semi-quantified **(B)**. **(C)** The mRNA level of RAGE in NRK-52E cells determined by qPCR. **(D)** The viability status of NRK-52E cells observed under a microscope. **(E)** Quantification of cell viability by CCK-8. The protein level of RAGE cleaved caspase 3 **(F)** was assessed by western blot, followed by semi-quantification **(G)**. One-way ANOVA was used to compare means between groups. Data are expressed as mean ± SEM. *p < 0.05, **p < 0.01, ***p < 0.001, ****p < 0.0001; FPS, FPS-ZM1.

Nephroprotection of RAGE deficiency was verified on NRK-52E cells with FPS-ZM1, a specific inhibitor of RAGE (Deane et al., 2012). First, we confirmed upregulation of RAGE induced by cisplatin and inhibitory effect of FPS-ZM1 on RAGE at mRNA and protein levels in NRK-52E cells (Figures 6A–C). Then, we showed that FPS-ZM1 at no more than 20 μM was not toxic to NRK-52E cells (Supplementary Figure S2); cisplatin reduced cell viability, which was visibly and statistically reversed by FPS-ZM1 (Figures 6D,E). The inhibitory effect of FPS-ZM1 on apoptosis was further illustrated by the modification in the expression level of cleaved Caspase-3 protein (Figures 6F,G). These results are in agreement with the aforementioned findings of animal experiments, further suggesting that inhibition of RAGE directly attenuates the toxicity of cisplatin on renal TECs.

### FPS-ZM1 Diminished Cisplatin-Induced Lipid Accumulation and FAO Impairment of NRK-52E Cells

**FIGURE 7:**
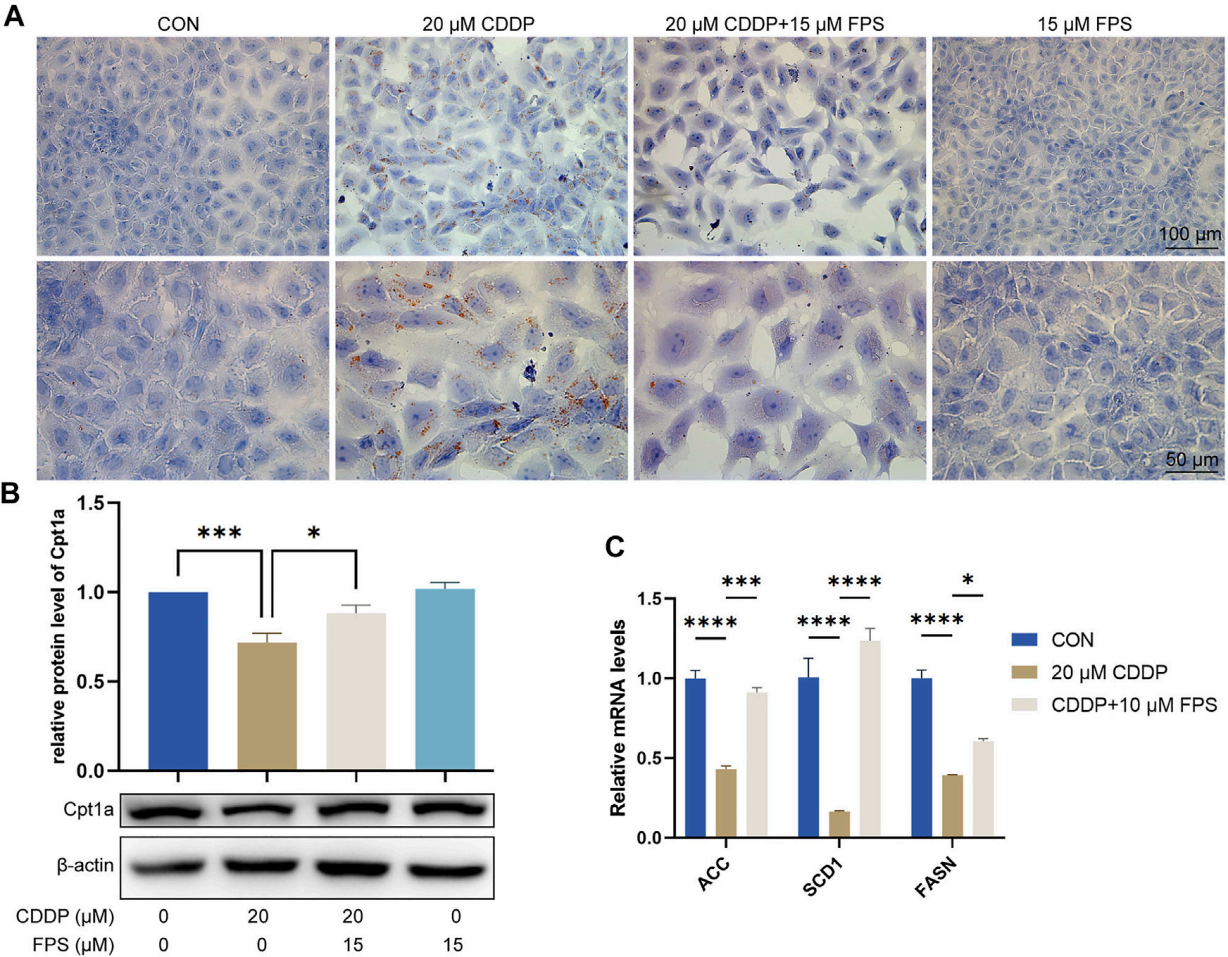
FPS-ZM1 diminished cisplatin-induced renal lipid accumulation and FAO impairment. **(A)** Photomicrographs illustrated lipid deposition in NRK-52E cells by using Oil red O staining. **(B)** Protein expression profiling of Cpt1a by western blot. One-way ANOVA was used to compare means between groups. **(C)** The mRNA levels of fatty acid synthesis-related genes ACC, SCD1, and FASN determined by qPCR. Two-way ANOVA was used to compare means between groups. Data are expressed as mean ± SEM. Scale bar: 100 or 50 μm as indicated in the figure. *p < 0.05, ***p < 0.001, ****p < 0.0001.

To evaluate and validate the effect of FPS-ZM1 on lipid metabolism in NRK-52E cells, we performed Oil red O staining and observed that cisplatin prominently elicited cellular lipid deposition, which was significantly restrained by FPS-ZM1 (Figure 7A). This is consistent with the *in vivo* findings. Further qPCR results showed that cisplatin significantly inhibited FAO in NRK-52E cells, while FPS-ZM1 blocked this process, thereby inhibiting lipid deposition (Figure 7B). In addition, we also evaluated the synthesis rate of free fatty acid. Unexpectedly, cisplatin significantly halted the fatty acid synthesis capacity of NRK-53E cells indicated by reduced mRNA levels of ACC, SCD1 and FASN genes, while FPS-ZM1 dramatically restored their mRNA levels (Figure 7C). Collectively, FPS-ZM1 diminished cisplatin-induced lipid accumulation and FAO impairment of NRK-52E cells.

## Discussion

Cisplatin is a widely used anticancer agent, yet frequently accompanied by nephrotoxicity with elusive mechanisms that may involve mitochondrial damage, impaired FAO, oxidative stress, endoplasmic reticulum stress, inflammation, apoptosis, necroptosis, etc. RAGE is a multiligand pattern recognition receptor, engaged in the regulation of inflammation, apoptosis, and FAO, and expressed in multiple cells, including renal TECs (Morcos et al., 2002). Previous studies have showed that RAGE involves epithelial-mesenchymal transition of renal TECs and adriamycin-induced glomerulosclerosis (Oldfield et al., 2001; Guo et al., 2008), which led us to decipher the role of RAGE in cisplatin nephrotoxicity.

The present study revealed for the first time that RAGE knockout significantly attenuated cisplatin-induced nephrotoxicity, as evidenced by reduced renal apoptosis, inflammation, lipid accumulation, as well as restored mitochondrial homeostasis and FAO. *In vitro* experiments showed that the RAGE-specific inhibitor FPS-ZM1 counteracted the inhibitory effect of cisplatin on cell viability and FAO of the rat renal TEC line NRK-52E. These findings elaborate the essential role of RAGE in cisplatin nephrotoxicity, suggesting that RAGE inhibition holds promise as a new therapeutic strategy to mitigate cisplatin nephropathy.

Renal TECs are highly susceptible to apoptosis, which renders apoptosis an important mechanism of cisplatin nephrotoxicity (Havasi and Borkan, 2011). Pharmacological inhibition or gene knockout of RAGE could alleviate apoptosis in renal cells in a diverse range of settings (Zhou et al., 2012; Hagiwara et al., 2018; Mao et al., 2018). We therefore investigated if RAGE deletion could attenuate cisplatin-induced apoptosis in renal TECs. Both *in vivo* and *in vitro* experiments suggested that RAGE suppression restored cisplatin-induced apoptosis in renal TECs.

Cisplatin leads to renal inflammation (Hsing et al., 2021; Imig et al., 2021). NF-κB is a canonical signal that mediates renal inflammation (Sanz et al., 2010; Ozkok et al., 2016; Liu et al., 2017). RAGE can mediate inflammatory signaling by regulating NF-κB (Volz et al., 2012; Ali et al., 2015; Dwir et al., 2020). Our results showed that RAGE deficiency reduced cisplatin-activated NF-κB and decreased the transcription of NF-κB downstream pro-inflammatory factors TNF-α and IL-6, chemokine MCP-1, and COX-2, as well as macrophage infiltration in the kidney. These results suggest that RAGE/NF-κB signaling may mediate cisplatin-induced renal inflammation.

Mitochondrial homeostasis is essential for the survival of renal TECs (Yu et al., 2018; Wang et al., 2020), whereas prone to impairment by cisplatin (Szeto, 2017; Lu et al., 2020). Studies indicate that modulation of RAGE improves mitochondrial injury (Yu et al., 2017; Mao et al., 2018; Syed et al., 2020). We revealed that RAGE knockout reversed the decrease in mitochondrial number of renal TECs caused by cisplatin. These data further suggest a link between RAGE and mitochondrial homeostasis.

Apart from being a source of energy, fatty acids are also engaged in the formation of mitochondrial membrane phospholipids. Calcium-independent Phospholipase A2γ can repair damaged mitochondrial membrane phospholipids by hydrolyzing damaged acyl chains to make them re-esterify with fatty acids and thus maintain mitochondrial survival and function, including FAO (Elimam et al., 2013).

FAO is the primary source of energy for renal TECs (Kang et al., 2015), but susceptible to inhibition by cisplatin, which leads to failed access to sufficient energy for cells and ultimately causes lipid accumulation and cellular insult (Jang et al., 2020). Previous studies suggest that RAGE is implicated in the regulation of FAO (Wan et al., 2020). We revealed that knockout of RAGE halted cisplatin-induced lipid accumulation and FAO impairment in murine kidney, which was further confirmed by *in vitro* experiments. Given that FAO mainly occurs in mitochondria, it is consistent with the aforementioned results and further elaborates a tight link between RAGE and mitochondria. Finally, we unexpectedly identified a restorative effect of FPS-ZM1 on fatty acid synthesis capacity. Considering that the predominant energy source of renal TECs is lipids rather than glucose (Kang et al., 2015), NRK-52E cells may need to convert glucose to lipids before oxidizing it for energy supply. This may explain why FPS-ZM1 attenuates lipid accumulation in cells when restores fatty acid synthesis, i.e., it facilitates both the supply and utilization of the energy substance, lipids. Of course, further studies are warranted to confirm this.

In conclusion, RAGE deficiency ameliorates cisplatin nephrotoxicity by reducing apoptosis, inflammation, and restoring FAO in TECs. It suggests that RAGE may serve as a promising therapeutic target for the treatment of cisplatin nephrotoxicity.

## Data Availability

The original contributions presented in the study are included in the article/Supplementary Materials, further inquiries can be directed to the corresponding author.
