# Corrigendum: Receptor of advanced glycation end products deficiency attenuates cisplatin-induced acute nephrotoxicity by inhibiting apoptosis, inflammation and restoring fatty acid oxidation

**DOI:** 10.3389/fphar.2022.1022539

**Published:** 2022-11-01

**Authors:** Qiang Wang, Yuemei Xi, Binyang Chen, Hairong Zhao, Wei Yu, De Xie, Weidong Liu, Furong He, Chenxi Xu, Jidong Cheng

**Affiliations:** ^1^ Department of Internal Medicine, Xiang’an Hospital of Xiamen University, School of Medicine, Xiamen University, Xiamen, China; ^2^ Xiamen Key Laboratory of Translational Medicine for Nucleic Acid Metabolism and Regulation, Xiamen, China

**Keywords:** rage, cisplatin-induced nephrotoxicity, apoptosis, inflammation, fatty acid oxidation

In the published article, there was an error in affiliation **1**. Instead of “Department of Endocrinology, Xiang’an Hospital of Xiamen University, Xiamen, China”, it should be “Department of Internal Medicine, Xiang’an Hospital of Xiamen University, School of Medicine, Xiamen University, Xiamen, China”.

In the published article, the reference **Elimam et al.** was not cited. The citation has now been inserted in **Discussion**, Paragraph 6, and now reads:

“Apart from being a source of energy, fatty acids are also engaged in the formation of mitochondrial membrane phospholipids. Calcium-independent Phospholipase A2γ can repair damaged mitochondrial membrane phospholipids by hydrolyzing damaged acyl chains to make them re-esterify with fatty acids and thus maintain mitochondrial survival and function, including FAO (Elimam et al., 2013).”

The authors apologize for this error and state that this does not change the scientific conclusions of the article in any way. The original article has been updated.
