# Supplementary material for: Receptor of Advanced Glycation End Products Deficiency Attenuates Cisplatin-Induced Acute Nephrotoxicity by Inhibiting Apoptosis, Inflammation and Restoring Fatty Acid Oxidation
Source: Front Pharmacol. 2022 May 30;13:907133. doi: 10.3389/fphar.2022.907133 (PMC9196246; doi:10.3389/fphar.2022.907133)

Supplementary Figure 1. Genotype identification of mice. Genotype of RAGE-/- mice was confirmed by RT-PCR (**A**) and western blot (**B**). KO, RAGE knockout; HT, Heterozygote.


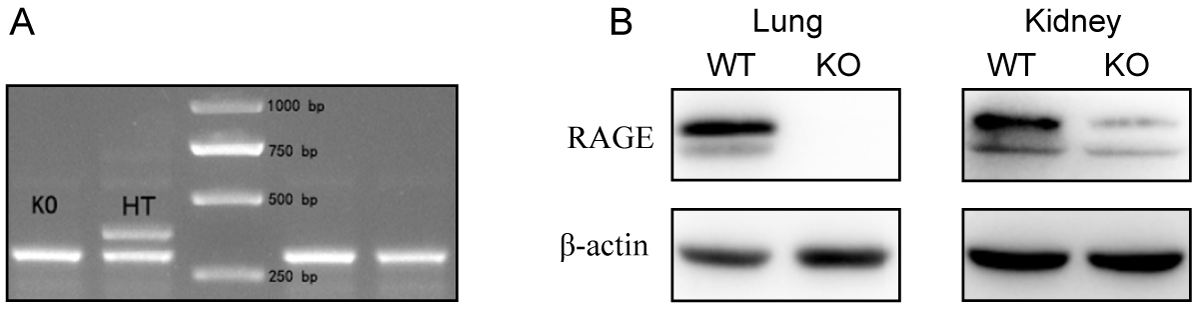


Supplementary Table 1. primer sequences

| Genes | Primer sequence (5’→3’) |
| --- | --- |
| mouse ACTB | F: GGCTGTATTCCCCTCCATCG  R: CCAGTTGGTAACAATGCCATGT |
| mouse KIM-1 | F: ACATATCGTGGAATCACAACGAC  R: ACAAGCAGAAGATGGGCATTG |
| mouse TNF-α | F: TTCTCATTCCTGCTTGTGG  R: TTGGGAACTTCTCATCCCT |
| mouse COX-2 | F: CACAGGATTTGACCAGTATAAGTG  R: GTCAGAAATTCAGGTGTAGTACAG |
| mouse MCP-1 | F: CAACTCTCACTGAAGCCAG  R: TTAACTGCATCTGGCTGAG |
| mouse IL-6 | F: TACCACTTCACAAGTCGGA  R: AATTGCCATTGCACAACTC |
| mouse Bcl-2 | F: TGACTGAGTACCTGAACCG  R: TAGTTCCACAAAGGCATCC |
| mouse BAX | F: CTGCAGAGGATGATTGCTG  R: ATCAGCAAACATGTCAGCT |
| mouse Cpt1a | F: GGTCTTCTCGGGTCGAAAGC  R: TCCTCCCACCAGTCACTCAC |
| mouse mtND1 | F: ATCCTCCCAGGATTTGGAAT  R: ACCGGTAGGAATTGCGATAA |
| mouse PGC-1α | F: AGTCCCATACACAACCGCAG  R: CCCTTGGGGTCATTTGGTGA |
| rat ACTB | R: CTAGGAGCCAGGGCAGTAATCT  F: AAGACCTCTATGCCAACACAGTG |
| rat ACC | F: GAGTCTGGCTACTACTTGGA  R: ATACGCCTGAAACATGATCTG |
| rat SCD1 | F: ACATGCTCCAAGAGATCTCC  R: GTCCATTCTGCAGGTTTCC |
| rat FASN | F: TTAATTGGCTCCACCAAATCC  R: GGATAACAGCACCTTGGTC |

Supplementary Figure 2. Effect of FPS-ZM1 on cell viability determined by CCK-8. One-way ANOVA was used to compare means between groups. Data are expressed as mean ± SEM. *****P* < 0.0001; FPS, FPS-ZM1.


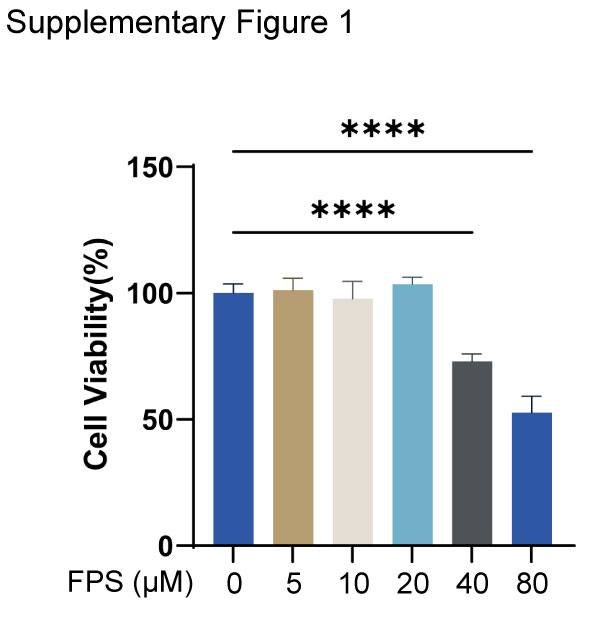

Supplement: Supplementary file 1 [file DataSheet1.docx]
